# Are serum amyloid A or D-lactate useful to diagnose synovial contamination or sepsis in horses?

**DOI:** 10.1136/vr.104386

**Published:** 2017-10-19

**Authors:** Claire S Robinson, Ellen R Singer, Martina Piviani, Luis M Rubio-Martinez

**Affiliations:** 1 Department of Equine Clinical Science, Institute of Veterinary Science, University of Liverpool, Wirral, UK; 2 Department of Small Animal Clinical Science, Institute of Veterinary Science, University of Liverpool, Wirral, UK

**Keywords:** saa, equine, synovial sepsis, septic arthritis, D-lactate

## Abstract

Synovial sepsis in horses is life threatening and accurate diagnosis allowing prompt treatment is warranted. This study assessed the diagnostic value of serum amyloid A (SAA) and D-lactate in blood and synovial fluid (SF) as diagnostic markers of synovial sepsis in horses and correlated them with total nucleated cell count (TNCC), percentage of neutrophils (%N) and total protein (TP) in SF. Blood and SF SAA and D-lactate concentrations were determined in a case–control observational study including 112 horses (38 with synovial contamination or sepsis (SCS), 66 with non-septic intra-synovial pathology (NSISP) and 8 controls). Blood and SF SAA were significantly higher in SCS than in NSISP and control horses. SAA values were similar in NSISP and control horses. SF SAA was moderately correlated with synovial TNCC, TP and blood SAA. Blood and SF SAA were 82.4 per cent and 80 per cent sensitive and 88.9 per cent and 73 per cent specific for diagnosis of SCS, with cut-off values of 60.7 and 1.14 µg/ml, respectively. Blood and SF D-lactate concentrations were not significantly different between groups. This study shows that blood and SF SAA concentrations can aid to distinguish SCS from non-septic synovial pathology; however, D-lactate was not useful.

Bacterial contamination of synovial cavities leading to synovial infection is life threatening for horses but early diagnosis and treatment can improve survival.^1 2^ The most specific diagnostic tests are microbiological culture from synovial fluid (SF) or visualisation of intracellular bacteria on SF smear, but their sensitivity is low. Bacterial culture is positive in only 32 per cent of septic SF samples.^3^ Total nucleated cell count (TNCC), percentage of neutrophils (%N) and total protein (TP) in SF are used by most practitioners to diagnose and monitor synovial sepsis; however, their concentrations can be equivocal and are affected by common treatments such as through-and-through synovial lavage, arthroscopic synovial lavage and intra-synovial amikacin.^4–6^ Therefore, early diagnosis can be difficult and investigation into other indicators of synovial sepsis is warranted.

Serum amyloid A (SAA) is an acute-phase protein synthesised predominantly by the liver but also by extrahepatic sources including synovial membrane.^7^ Plasma SAA concentrations are negligible in healthy horses but rapidly increase following tissue injury, infection and inflammation, providing an objective reflection of the extent of disease.^8^ Synovial SAA concentrations were increased in a limited number of horses with experimental and naturally occurring septic arthritis^9 10^ and were not affected by common treatments for septic arthritis such as intra-articular medication or surgical lavage.^4–6^ Measurement of SAA is affordable and widely available and could be useful to aid rapid and accurate diagnosis of synovial sepsis.

D-lactate is a stereoisomer of mammalian L-lactate, produced by bacterial fermentation and not produced by mammalian tissue.^11^ The presence of D-lactate in body fluids has been reported to indicate bacterial contamination or infection.^12 13^ Bacterial arthritis has been associated with elevated SF D-lactate concentrations in humans.^14^ In horses, D-lactate concentrations in peritoneal fluid were elevated in horses with septic peritonitis or gastrointestinal rupture in comparison with horses with non-strangulating obstructions of the gastrointestinal tract^15^; however, D-lactate concentrations in SF of horses have not previously been investigated.

The study’s aim was to evaluate the diagnostic value of SAA and D-lactate concentrations in blood and SF from horses with synovial contamination and sepsis and to assess the correlation between these parameters and SF TNCC, %N and TP. The authors’ objectives were to measure SAA and D-lactate concentrations in blood and SF from horses with synovial structures which were normal (control), had non-septic, intra-synovial pathology (NSISP) or had synovial contamination or sepsis (SCS).

The authors’ hypotheses were that horses with a contaminated or septic synovial structure would have higher blood and SF SAA and D-lactate concentrations than horses with healthy joints or NSISP. Further to this, the authors hypothesised that blood and SF SAA and D-lactate would be positively correlated with SF TNCC, %N and TP.

## Materials and methods

### Inclusion criteria

A case–control observational study was performed. Horses presented for lameness investigation, intra-synovial surgery, SCS, and horses presented for euthanasia for non-orthopaedic, non-inflammatory conditions at the Philip Leverhulme Equine Teaching Hospital (University of Liverpool) between October 2013 and January 2015 were recruited following owner consent.

### Study groups

Horses were categorised into three groups: control, NSISP or SCS. Definition of SCS was based on previous reports.^2 16–18^ Cases were included in the SCS group if they met ≥1 of the following criteria: elevation of all SF parameters (TNCC >20 x 10^9^ cells/l, %N>80 per cent and TP >30 g/l)^16^; direct communication of a wound with the synovial cavity; positive bacteriological culture and/or visualisation of intracellular bacteria on an SF smear. In cases with previous intra-articular sampling or medication, at least one additional criterion apart from the elevated SF parameters had to be met. The NSISP group included horses with intra-synovial conditions comprising osteoarthritis, osteochondrosis, intra-articular fracture and non-septic tenosynovitis. None of the horses in this group had any known septic or infectious condition. The control group included SF samples from horses with no clinically evident inflammatory disease or lameness that were euthanased or horses that had a lameness investigation with a negative response to intra-synovial analgesia of the sampled joint. The following values were considered as normal ranges for SF analysis: TNCC ≤3.5 x 10^9^ nucleated cells/l with <20 %N and TP < 25 g/l^19^ ^20^.

### Sample collection and processing

For horses with SCS, samples were obtained at admission or at the start of arthroscopy or tenoscopy. For the NSISP group, samples were obtained during lameness investigation as part of diagnostic work-up or at the start of arthroscopy or tenoscopy. For the control group, samples were collected during intra-synovial anaesthesia or at the time of euthanasia.

SF samples were collected aseptically. TP concentration was measured on SF without anticoagulant with a refractometer (Master Refractometer, Atago, Japan). One to two millilitres of the sample were transferred into both an EDTA and lithium heparin vacutainer (BD Vacutainer, Belliver Industrial Estate, Plymouth, UK) for cytological examination and SAA, and for D-lactate determinations, respectively. Blood samples were collected from the jugular vein in plain tubes with clot activator (BD Vacutainer) for serum SAA analysis and in lithium heparin tubes for plasma D-lactate measurement.

SF TNCC was determined on the EDTA sample using an automated cell counter (VetScan HM5, Abaxis Europe, Bunsenstrasse 9-11, Griesheim, 64347, Germany). All blood and SF samples were centrifuged within 10 minutes of collection at 4000 g for 15 minutes at 4⁰C. Serum, plasma and SF supernatant were stored at −80⁰C until analysed. Maximum storage time was one year, during which SAA has been shown to remain stable.^21^ Samples were analysed in one batch by the same person to limit variability and inaccuracies in results due to repeated freeze-thaw cycles.

### SAA quantification

Samples were taken from the −80°C freezer and thawed at room temperature immediately before processing. SAA concentration was determined using an automated chemistry analyser with a human SAA turbidometric immunoassay (Eiken LZ SAA test, Eiken Chemical, Mast Group, Bootle, Merseyside, UK) as previously described in horses.^6 9 22^ A value of 0 was used for concentrations below the limit of detection (0.5 µg/ml).

### D-lactate quantification

D-lactate concentrations were determined using the lactate dehydrogenase technique with a commercial D-lactate analysis kit and Megacalc software (Megazyme International, Wicklow, Ireland). This technique has been described for D-lactate concentrations in human blood and peritoneal fluid,^23^ human SF^24^ and equine peritoneal fluid.^15^ SF and blood samples defrosted at room temperature and then centrifuged using polyethersulfone ultrafiltration membranes (Fisher Scientific, Leicester, UK) at 4000 g for 10 minutes to improve test accuracy.^23^ A value of 0 was used for measurements below the limit of detection (0.21 µg/ml).

### Statistical analysis

Breed and number of horses of joints affected are presented as number of observations. Normality of data was assessed by Kolmogorov-Smirnov statistical test. Data were non-normally distributed despite transformations and were analysed with Kruskal-Wallis and post hoc Dunn’s tests. Descriptive data are presented as median and range. Correlations between variables were investigated using Spearman’s rank correlation coefficient test and 95 per cent CIs calculated using bootstrapping and the bias-corrected and accelerated method. A receiver operating characteristics (ROC) curve was generated to allow calculation of the sensitivity, specificity and cut-off value for SAA in blood and SF for the diagnosis of synovial sepsis. Statistical analysis was performed with the commercial software IBM SPSS Statistics V.2 (IBM United Kingdom Limited, Portsmouth, UK). For all analyses, P<0.05 was considered significant.

### Ethical approval

Ethical approval for the study was obtained from the Institute of Veterinary Science University of Liverpool Ethics Committee (VREC175).

## Results

In total, 112 horses were included: SCS (n=38), NSISP (n=66) and controls (n=8). Age of horses did not differ significantly between groups (median 10 years (14 days to 23 years)). Distributions of breed and affected synovial structures are listed in Table 1. Gender distribution included 43 mares, 3 stallions and 66 geldings. None of the horses in the NSISP or control groups had received intra-articular medications before surgery. In the SCS group, five horses had sustained a synoviocentesis of the affected synovial structure and three of those horses had received intra-articular antimicrobials before referral by the referring veterinarian. Twenty-one horses had SF samples submitted for bacterial culture and seven horses had positive results. Bacteria identified included oxacillin-sensitive *Staphylococcus* species (3/7), *Bacillus* species (1/7), *Enterococcus faecalis* (1/7), non-haemolytic *Escherichia coli* (1/7) and *Actinobacillus suis* (1/7). One sample which cultured *Staphylococcus* species also cultured *Streptococcus dysgalactiae* var. *equimilis*.

**TABLE 1:** Distribution of breeds and synovial structures involved from a total of 112 horses included in the study.

Breed of horse	Horses (n)	Synovial structure affected	Horses (n)
Thoroughbred and thoroughbred cross	23	Digital flexor tendon sheath	25
Irish draft and Irish draft cross	15	Metacarpophalangeal/ metatarsophalangeal joint	22
Warmblood	10	Tarsocrural joint	17
Cob	9	Radiocarpal joint	9
Welsh pony	9	Navicular bursa	9
Irish sports horse	6	Distal interphalangeal joint	5
Hanoverian	6	Femoropatellar joint	4
Connemara	3	Middle carpal joint	4
Belgian warmblood	3	Calcaneal bursa	4
Appaloosa	2	Sheath of extensor carpi radialis	3
Other/unknown breed	26	Carpal sheath	2
		Elbow joint	2
		Bicipital bursa	2
		Tarsal sheath	2
		Tarsometatarsal joint	1
		Proximal interphalangeal joint	1

Horses with SCS had significantly higher values of SF and blood SAA than horses with NSISP (P=0.03 and 0.02, respectively) and higher SF SAA than the control group (P=0.009); however, no differences were found between NSISP and control groups. Concentrations of D-lactate in blood and SF did not differ among groups (P values: 0.639 and 0.268, respectively) (Table 2).

**TABLE 2:** Concentrations of serum amyloid A (SAA) in synovial fluid and blood of horses with synovial sepsis and contamination (SCS), non-septic intra-synovial pathology (NSISP) and control groups.

	SCS	NSISP	Control
Horses (n)	38	66	8
Synovial fluid SAA (µg/ml)	39.2^a^ (0–368.9)	0^b^ (0–29.7)	0^b^ (0–0.8)
Blood SAA (µg/ml)	275.5^a^ (0–1421.8)	0.5^b^ (0–17)	0^b^ (0–0)
Synovial fluid D-lactate (µg/ml)	30 (0–640)	20 (0–84)	35 (0–80)
Blood D-lactate (µg/ml)	12.5 (0–90)	10 (0–70)	20 (0–50)
Synovial fluid total nucleated cell count (cells/l) Median (range)	42.8 (2.7–234)^a^	0.39 (0–14)^b^	0.5 (0.08–3.3)^b^
Synovial fluid % neutrophils Median (range)	84 (42-94)^a^	55 (0–88)^b^	12 (0–20)^b^
Synovial fluid total protein (g/l) Median (range)	44 (23-84)	14 (0.8–44)	11.5 (6-19)

The SAA concentrations are reported as median (range). Within a row, different superscript letters denote significant differences between groups (P<0.05)

Synovial fluid SAA was positively correlated with SF TNCC (P≤0.001) and blood SAA (P=0.006). Blood SAA was also positively correlated with SF TNCC (P≤0.001) and SF TP (P=0.015) (Table 3).

**TABLE 3:** Spearman’s non-parametric correlations and 95% CIs between synovial fluid (SF) total nucleated cell count (TNCC), SF total protein (TP), SF serum amyloid A (SAA), SF D-lactate and blood SAA.

Variable correlation	Correlation coefficient	95% CI	P value
TNCC and SF SAA	0.639	0.220 to 0.862	0.014
TNCC and blood SAA	0.808	0.403 to 0.979	0.000
TP and blood SAA	0.631	0.285 to 0.800	0.015
SF SAA and blood SAA	0.693	0.363 to 0.867	0.006

Only significant correlations are included in the table (P<0.05)

**FIG 1:**
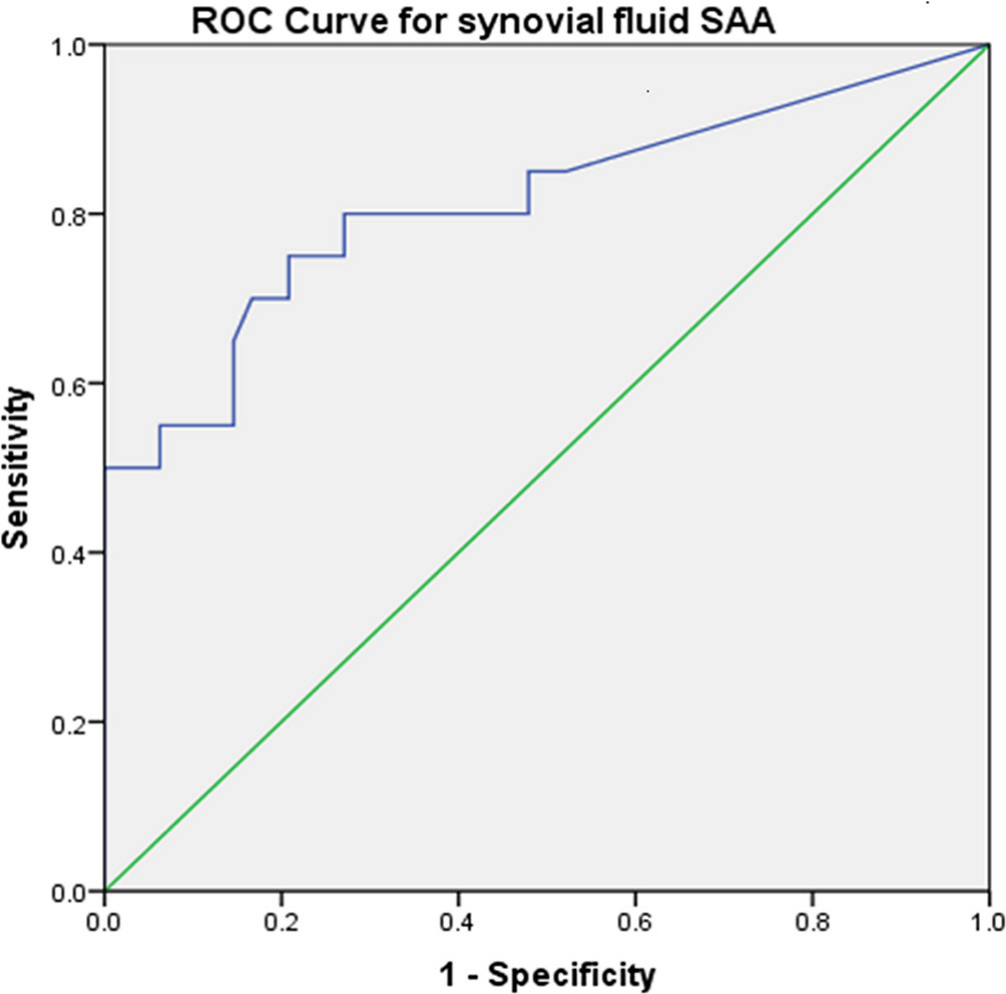
Receiver operating characteristics (ROC) curve for synovial fluid (SF) serum amyloid A (SAA) concentrations as a diagnostic marker for synovial sepsis or contamination in horses. Based on a cut-off value of 1.14 µg/ml for SF, SAA had a sensitivity of 80% and specificity of 73% for the diagnosis of synovial contamination or sepsis.

**FIG 2:**
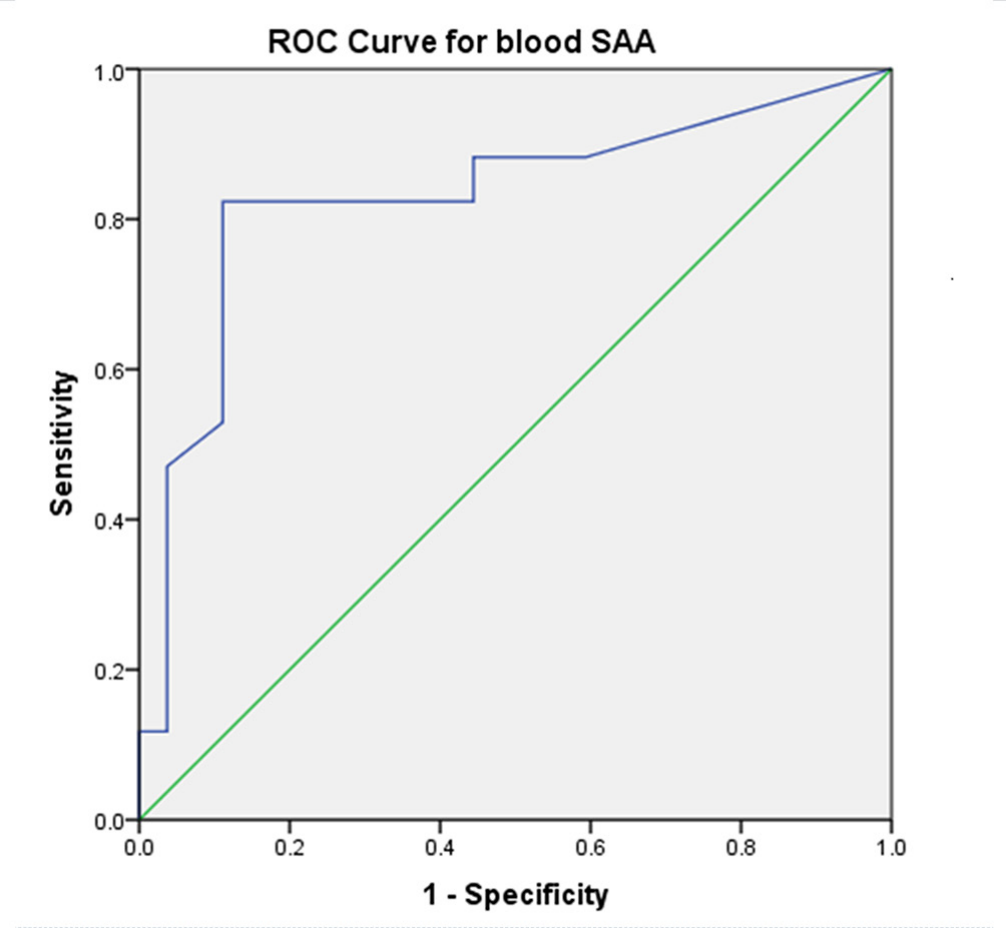
Receiver operating characteristics (ROC) curve for blood serum amyloid A (SAA) concentrations as a diagnostic marker for synovial sepsis or contamination in horses. Based on a cut-off value of 60.7 µg/ml for blood, SAA had a sensitivity of 82.4% and specificity of 88.9% for the diagnosis of synovial contamination or sepsis.

The ROC curve calculated for SF SAA provided a sensitivity of 80 per cent and specificity of 73 per cent for the diagnosis of SCS, based on an SF SAA cut-off value of 1.14 µg/ml (Figure 1). The ROC curve calculated for blood SAA provided a sensitivity of 82.4 per cent and specificity of 88.9 per cent based on a blood SAA cut-off value of 60.7 µg/ml (Figure 2).

## Discussion

This study indicates that SAA is elevated in the blood and SF of horses with SCS but not in horses with non-septic joint pathology or normal joints. SAA in blood and SF had moderate-to-high sensitivity and specificity for differentiating horses with SCS from horses with NSISP or horses with normal synovial structures in the authors’ population of horses. Therefore, SF and blood SAA could be used to aid diagnosis of SCS. On the contrary, levels of D-lactate were not useful for diagnosis of SCS.

Elevation of SF SAA in horses with SCS has been reported previously in a limited number of clinical^9^ and experimental^10^ cases of septic arthritis, with the study presented here being the first report on a large number of clinical cases. This study also demonstrates that blood SAA increases significantly in horses with SCS, in agreement with Ludwig and others,^10^ and that blood and SF concentrations of SAA are moderately correlated in horses. The majority of equine SAA is synthesised in the liver and systemic SAA can cross into synovial cavities^22^; however, in the presence of inflammatory or septic conditions, extrahepatic synthesis of SAA, including production by synoviocytes, may increase.^22^ Identification of individual SAA isoforms indicates specific cell-type origins of SAA production but this information is not provided by the human SAA turbidometric immunoassay used in this study. The moderate correlation between systemic and SF SAA concentrations and the higher systemic compared to articular levels of SAA may suggest an extra-articular source of SAA in cases with synovial sepsis. A study on human patients with articular pathologies concluded that SAA present in the SF was the result of passive diffusion from blood into the joint cavity, rather than from de novo production within the synovial structures.^25^ A systemic response characterised by increased blood SAA has been recognised after castration in horses with increased postoperative inflammation and/or infection at the castration site compared with horses with no surgical-site complications.^26^ Further work is warranted to investigate blood SAA as a marker for monitoring synovial sepsis in horses.

In the present study, blood and synovial SAA had moderate to high sensitivity (80–82.4 per cent) and specificity (73–88.9 per cent) for the diagnosis of SCS in their hospital population, which indicates their value as diagnostic markers for SCS. Direct extrapolation of these cut-off values to other equine hospitals or general equine populations should be made carefully as the current study has inherent biases. Due to the selective nature of the horse population, there is risk for an underestimation or, more likely, overestimation of the performance of the test.^27^ Further studies are warranted to assess the validity of synovial and blood SAA as a diagnostic or monitoring marker of the progression or resolution of synovial sepsis in the equine general population.

SF D-lactate has been successfully used to differentiate between septic (D-lactate >4.5 µg/ml) and non-septic synovial structures (D-lactate <4.5 µg/ml) in a human study.^14^ However, other studies have found equivocal results and concluded that D-lactate was not useful to diagnose bacterial synovitis.^24^ Some of the D-lactate concentrations in contaminated and septic cases in this study were similar to those in human studies^14 24^ and were higher than in the non-septic groups, although the differences were not significant. Differences in human versus animal species, bacterial populations and/or environmental conditions may also account for differences in D-lactate concentrations among studies. Anaerobic versus aerobic conditions increased D-lactate production.^28^ High SF D-lactate levels have been demonstrated previously in human SF from septic synovial structures which cultured *Staphylococcus aureus*, β-haemolytic *Streptococcus* species, *Staphylococcus epidermidis* 24 and *Serratia marcescens* or *Mycobacterium tuberculosis*.^29^ D-lactate production has also been described from the anaerobic order of *Lactobacillales* such as *Lactobacillus* species, *Leuconostoc* species, *Pediococcus* species and *Enterococcus* species.^28^ However, D-lactate production by other bacteria in SF is unknown. The limited number of positive cultures in this study precluded any further investigation in this regard. A recent report on bacterial isolates from equine synovial sepsis reported a low prevalence of known D-lactate-producing bacteria (11 per cent *S aureus,* 1 per cent *S* epidermis, 2 per cent β-haemolytic *Streptococcus* species, 7 per cent *Streptococcus* species, 6 per cent *Enterococcus* species),^30^ which could explain the limited diagnostic value of D-lactate observed in this study. Further work is required to investigate if D-lactate levels are correlated to the number and type of bacteria in the sample.

The two assays used in this study for SAA and D-lactate determination were selected based on previous work in horses. The turbidometric immunoassay is routinely used to measure SAA in equine blood and SF.^4–6 9 22^ Previous validation on equine samples showed coefficients of variation at high, intermediate and low concentrations of SAA of 2.1 per cent, 1.6 per cent and 24.4 per cent respectively for intra-assay variation; and 6.5 per cent, 4.6 per cent and 33.2 per cent respectively for inter-assay variation.^31^ The spectrophotometer assay is the most widely used method of determination of D-lactate levels in in human body fluids including SF^14 24^ and has been used recently on equine peritoneal fluid.^15^ However, use on equine blood or SF has not previously been performed to the authors’ knowledge.

In the authors’ hospital population, the majority of SCS cases were secondary to wounds that occurred while the horse was turned out at pasture. Although not recorded for this study, these cases typically present to their hospital >6–8 hours after the injury occurred. Concentrations of SAA in serum have been shown to take 4–8 hours to elevate in horses with lipopolysaccharide-induced arthritis,^22^ although peak values were not reached until later in the experimental septic arthritis model.^10^ The cut-off values observed in this study are within the range of those observed within the first 24 hours after inducing septic arthritis in horses.^10^ Effects of causative incident of sepsis, time to sampling or type of synovial structure involved (tendon sheath, bursae or joint) may have an impact of variability of SAA and D-lactate levels.

All horses included in this study were referred following diagnostic assessment and/or treatment administered by the referring veterinarian which may have affected results. The systemic administration of anti-inflammatories or antimicrobials may have affected the production and/or kinetics of blood or SF parameters such as SAA. In horses with experimentally induced septic arthritis, administration of phenylbutazone led to delayed onset of serum and SF SAA elevation.^10^ The majority of cases in this study received NSAIDs before referral and the impact of this on SAA levels in this study is unknown and warrants further investigation as this may have high relevance in the clinical setting. Previous studies have shown that arthrocentesis, intra-articular injection of saline or amikacin,^6^ arthroscopic lavage^4^ or through-and-through lavage^5^ did not increase SAA concentrations in blood or SF of healthy horses. The effects of intra-synovial diagnostic or therapeutic injections on concentrations of D-lactate are unknown; however, D-lactate is only produced by bacteria and all procedures are performed under sterile conditions, so the effect is expected to be minimal. In addition, the concentrations were not significantly different between groups, and therefore, the effect is unlikely to have an impact.

This study provides evidence that blood and SF SAA concentrations can be used to differentiate between septic or contaminated versus non-septic intra-synovial pathology or normal synovial cavities in their hospital population; however, SAA concentration should be interpreted as an adjunct diagnostic test. Horses with concurrent inflammatory conditions, including those with large wounds or extensive soft tissue trauma, may also have elevated SAA and therefore SAA results should be interpreted with caution. Similarly, blood contamination of SF samples could cause an increased SAA concentration in horses with elevated systemic SAA. Further work is required to investigate the value of blood SAA as a monitoring and prognostic marker of synovial sepsis in horses. The clinical advantage of SAA testing is the ability to provide additional evidence in cases with equivocal TNCC, percentage of neutrophils and TP. A recommended cut-off value to indicate synovial sepsis is 1.14 µg/ml for SF and 61 µg/ml for blood. Based on this study, D-lactate concentrations were not useful to diagnose synovial sepsis in horses presented with synovial sepsis. In conclusion, SAA concentrations in blood and synovial fluid are useful to aid the diagnosis of synovial sepsis.
